# Adjunctive Pharmacotherapy for Elective Direct Current Cardioversion in Patients With Atrial Fibrillation

**DOI:** 10.4021/cr198w

**Published:** 2012-07-20

**Authors:** Liam S. Hirt, Maya S. Gobin

**Affiliations:** a Royal United Hospital, Bath, England; b Health Protection Agency, Bristol, England

**Keywords:** Atrial fibrillation, Antiarrhythmic drugs, Renin-angiotensin system, Cardioversion, Cardiac electroversion, Flecainide

## Abstract

**Background:**

Direct current cardioversion (DCCV) can restore sinus rhythm in patients with atrial fibrillation (AF), but the long term efficacy is poor. Pharmacological therapies may improve the initial success of the procedure, but whether long term maintenance of sinus rhythm can be improved is unclear. The aim of this study was to evaluate which pharmacotherapies, including antiarrhythmic and renin-angiotensin-aldosterone system (RAAS) inhibiting drugs, most successfully promotes sinus rhythm after elective DCCV in unselected patients with atrial fibrillation.

**Methods:**

A retrospective cohort was to study of AF patients attending or DCCV between Jan 2010 and Feb 2012. The data were analysed using multivariate logistical regression models. Initial success of DCCV was the dependent variable in the first analysis. Maintenance of sinus rhythm at follow up was the dependent variable in the second analysis.

**Results:**

One hundred and thirty patients were included in the first analysis, and 71 patients were included in the second analysis. The only association observed was a positive association between flecainide and an increased odds of maintaining sinus rhythm at follow up (OR 2.14, SE ± 0.93, P = 0.02) .Other antiarrhythmic drugs and RAAS inhibiting drugs had no association with an increased odds of successful DCCV or maintenance of sinus rhythm thereafter.

**Conclusions:**

This is the first study to demonstrate an association between flecainide and a increased odds of maintaining sinus rhythm after DCCV in the long term. This warrants further research, and should be taken into account when choosing adjunctive antiarrhythmic therapy for elective DCCV for AF.

## Introduction

Atrial fibrillation (AF) is common with a prevalence between 0.4% and 1% in the general population [1]. Direct current cardioversion (DCCV) can restore sinus rhythm in patients with AF, and if sinus rhythm is maintained, there is an associated improvement in quality of life [2]. DCCV has an initial failure rate of around 25%, and 25% of patients will revert back to AF within 2 weeks of a successful procedure [3]. After 1 year, 70% may have reverted back to AF [4]. Maintenance of sinus rhythm may be improved by selection of appropriate patients [4-7], the use of antiarrhythmic drugs, or repeat DCCV [8]. Despite this, the long term success of the procedure remains poor.

There have been numerous studies of adjunctive pharmacological therapies to improve the initial success of DCCV. A number of antiarrhythmic drug have been shown to be of benefit [9-13]. What remains unclear is whether these drugs also promote maintenance of sinus rhythm in the early post-procedural period, or longer term [10, 14, 15]. More recently there has been evidence to suggest that inhibition of the renin-angiotensin-aldosterone system (RAAS) might not only improve the initial success rate of the procedure, but also subsequent maintenance of sinus rhythm [16, 17]. However, the evidence is conflicting and the studies often involved the use of concomitant antiarrhythmics [18-20].

The aim of this study was to determine which adjunctive pharmacological therapy, including antiarrhythmic drugs and RAAS inhibiting drugs, most successfully promotes DCCV and maintenance of sinus rhythm in unselected patients with AF.

## Methods

This study was a single centre retrospective observational cohort study. All patients with atrial fibrillation who attended our hospital for an elective DC cardioversion between January 2010 and February 2012 were included, unless hospital records were unobtainable. Patients were identified and hospital records scrutinised for relevant data. Data recorded for each patient included age, sex, previous AF, previous DCCVs, medications, echocardiographic measurements, initial success of procedure, time to follow up and maintenance of sinus rhythm at follow up. Initial success of the procedure was defined as achieving sinus rhythm from DCCV and maintaining sinus rhythm until discharge on the same day.

The data were analysed in two multivariate logistical regression models. In the first analysis, restoration of sinus rhythm by DCCV was the dependent variable. The independent variables were those factors known, or believed, to influence the success of DCCV and/or maintenance of sinus rhythm thereafter. These included pharmacological therapies and also patient characteristics such as age, left atrial diameter and previous AF. Those patients who had been successfully cardioverted to sinus rhythm, and had subsequently had follow up, were included in the second logistical regression analysis. In the second model, the dependent variable was maintenance of sinus rhythm at follow up. Again, the independent variables were those factors known or believed to influence the success of DCCV and/or the maintenance of sinus rhythm thereafter. Statistical significance for both analyses was inferred at the 0.05 level.

## Results

One hundred and forty one patients were eligible for inclusion. Hospital documentation was not available for 11 patients, and these were excluded from the analyses. Of the 130 patients that had a DCCV, sinus rhythm was successfully restored in 105 (81%). Seventy one patients (68%) had documented follow up after successful DCCV, and off these, 22 (31%) had maintained sinus rhythm. The mean length of follow up was 190 days. The baseline characteristics of all patients, and those that had follow up after successful DCCV, are summarised in Table 1. The cohort was predominantly male (75%) with a mean age of 66.7 years (SD ± 10.44 years). All patients were established on their drug regime for at least 3 weeks prior to DCCV and antiarrhythmic and RAAS inhibiting drugs were continued unchanged in the post procedural period.

**Table 1:** Baseline Characteristics

Patient Characteristics	All Patients		Patients with follow up	
	Mean	(SD)	Mean	(SD)
Mean age (years)	66.7	(± 10.44)	66.82	± 9.45
Mean LA diameter (cm)	4.46	(± 0.68)	4.47	± 0.74

	n/N	(%)	n/N	(%)
Male	98/130	(75%)	52/71	(73%)
Previous DCCVs				
0	107/130	(82%)	57/71	(80%)
1	20/130	(15%)	11/71	(15%)
2	2/130	(2%)	2/71	(3%)
3	1/130	(1%)	1/71	(1%)
LV Systolic Impairment				
None	102/130	(79%)	55/71	(77%)
Mild	13/130	(10%)	9/71	(13%)
Moderate	11/130	(8%)	5/71	(7%)
Severe	4/130	(3%)	2/71	(3%)

Medication Usage	n/N	(%)	n/N	(%)
ACE inhibitors	48/130	(37%)	25/71	(35%)
ARBs	16/130	(12%)	7/71	(10%)
Amiodarone	13/130	(10%)	7/71	(10%)
Flecainide	14/130	(12%)	11/71	(15%)
Sotalol	4/130	(3%)	1/71	(1%)
Beta Blockers	96/130	(74%)	53/71	(75%)
Calcium Channel Blockers (non-DHP)	12/130	(9%)	7/71	
Calcium Channel Blockers (DHP)	15/130	(12%)	5/71	(7%)
Aldosterone antagonists	8/130	(6%)	5/71	(7%)
Digoxin	17/130	(13%)	9/71	(13%)

Outcomes	n/N	(%)	n/N	(%)
Sinus Rhythm	105/130	(81%)	22/71	(31%)

ACE: Angiotensin converting enzyme; ARB: Angiotensin receptor blocker; DCCV: Direct current cardioversion; DHP: Dihydropyridine; LA: Left atrium; LV: Left ventricle; N: Total number of patients; n: Number of patients with characteristic; SD: Standard deviation.

All 130 patients were included in the first analysis. In this analysis initial success of DCCV was the dependent variable. The results of the first logistical regression model are shown in Table 2. There were no significant associations between initial success of DCCV and the independent variables.

**Table 2:** Multivariate Logistical Regression Analysis With Successful DCCV as the Dependent Variable

Independent variables	Odds Ratio	Standard Error	P
RAAS inhibition	-0.11828	± 0.500	0.8131
Age > 65	0.96359	± 0.5630	0.0870
LA diameter	-0.10351	± 0.37118	0.7803
Flecainide	0.07556	± 1.03372	0.7803
Amiodarone	-0.05231	± 0.84642	0.9507
Sotalol	-2.51290	± 1.35484	0.0636
Beta Blocker	0.39456	± 0.53711	0.4626
Previous AF	0.84147	± 0.74584	0.2592

DCCV: Direct current cardioversion; LA: Left atrium; AF: Atrial fibrillation; RAAS: Renin-angiotensin-aldosterone system.

The seventy one patients that had follow up after successful DCCV and were included in the second regression model. In this analysis maintenance of sinus rhythm at follow up was the dependent variable. The results of the second logistical regression model are shown in Table 3. Only flecainide had a statistically significant association with maintenance of sinus rhythm at follow up (OR 2.14, SE ± 0.93, P = 0.02). Of the 14 patients taking flecainide, 11 had follow up and 5 had maintained sinus rhythm.

**Table 3:** Multivariate Logistical Regression Analysis With Maintenance of Sinus Rhythm at Follow up Following Successful DCCV as the Dependent Variable

Independent variables	Odds Ratio	Standard Error	P
RAAS inhibition	-0.03084	± 0.38379	0.9360
Age > 65	-0.15822	± 0.39373	0.6878
LA diameter	0.12418	± 0.28257	0.6603
Flecainide	2.14121	± 0.93122	0.0215
Amiodarone	0.09986	± 0.61899	0.8718
Sotalol	-2.04433	± 1.3987	0.1439
Beta Blocker	-0.11358	± 0.44340	0.7978
Previous AF	-0.44791	± 0.54313	0.4096

Boldface: Statistically significant; DCCV: Direct current cardioversion; LA: Left atrium; AF: Atrial fibrillation; RAAS: Renin-angiotensin-aldosterone system.

## Discussion

The main finding of this study was that flecainide therapy established prior to, and continued after, elective DCCV in unselected patients with AF, is associated with approximately a doubling of the odds of remaining in sinus rhythm at follow up. This is the first time an association has been shown between flecainide therapy and maintenance of sinus rhythm after DCCV in the longer term. A prior study with much a much shorter follow up period (4 weeks) failed to show an association between flecainide and maintenance of sinus rhythm [15].

Other antiarrhythmic drugs and RAAS inhibiting drugs had no association with successful DCCV or subsequent maintenance of sinus rhythm in this study. Amiodarone has previously been shown to increase the odds of a successful procedure compared to rate controlling medications in a number of trials, and there is some evidence to suggest that amiodarone also promotes maintenance of sinus rhythm over the subsequent 8 weeks [10]. The results presented here do not support the previous findings. This may be for a number of reasons. Firstly, this study involved an unselected cohort of patients. The benefit of amiodarone observed in small controlled trials may not directly translate into a benefit for unselected patients. Secondly, amiodarone is associated with potentially serious side effects, and non-compliance or discontinuation of amiodarone after DCCV is likely to have been more common in this cohort than in a controlled trial.

RAAS inhibition may reduce recurrence of AF after DCCV through a number of mechanisms, including attenuating changes in cardiac structure and function [21], preventing left atrial dilation, preventing atrial fibrosis, and preventing slowing of conduction velocities [22]. Which are independent of haemodynamic effects [23]. Despite these potential mechanisms there was no association between RAAS inhibiting drugs and maintenance of sinus rhythm in the current study. This finding is supported by a previous retrospective study [16]. Previous studies showing an association between maintenance of sinus rhythm and RAAS inhibition have also included antiarrythmic drugs [20]. Studies without concomitant antiarrythmics have failed to demonstrate the association [17, 24]. There are a number of possible explanations for the apparent discrepancy. The study presented here investigated RAAS inhibitors as an entire drug class. It is possible that some RAAS inhibiting drugs may have specific or exaggerated actions compared to other drugs of the same class, or have specific synergisms with antiarrhythmic drugs, but this remains to be tested.

The study presented here has a number of limitations. The observational nature of the current study means there may be a number of unknown and therefore uncontrolled confounding factors. However, the variables known to be associated with successful DCCV were included in the regression analysis, mitigating them as confounders. The observational nature of the study and the unselected cohort of patients enable a true reflection of the atrial fibrillation population, making the observations widely applicable. Follow up was incomplete, as would be expected with a retrospective analysis, and it is not clear whether those without follow up were more likely to have maintained sinus rhythm or not. Compliance with pharmacological therapy was not measured, and although antiarrhythmic and RAAS inhibiting medications were continued after DCCV, data concerning compliance. This may have implications for the results, as drugs such as amiodarone have potentially serious long term side effects, and are probably more likely to be discontinued that other well tolerated medications. However, this is a reflection of real clinical practise and does not affect the validity of the observations from the study presented here.

The current study has clear implications for clinical practise and future research. Further studies may be able address the limitations of the study presented here, and confirm the results in a larger cohort of patients. This would further define the role of pharmacological therapies in the maintenance of sinus rhythm after DCCV aiding clinical decision making. Currently, this is the only study showing an association between flecainide therapy and long term maintenance of sinus rhythm after elective DCCV for AF. There may be significant benefit in the longer term when compared to other antiarrhythmics and this should be taken into account by physicians when choosing adjunctive antiarrhythmics for DCCV.
